# Improving the future of clinical trials and translation of mesenchymal stromal cell therapies for neonatal disorders

**DOI:** 10.1093/stcltm/szae060

**Published:** 2024-08-09

**Authors:** Yun Sil Chang, Misun Yang, So Yoon Ahn, Se In Sung, Won Soon Park

**Affiliations:** Department of Pediatrics, Samsung Medical Center, Sungkyunkwan University School of Medicine, Seoul, Korea; Department of Health Sciences and Technology, Samsung Advanced Institute for Health Sciences and Technology (SAIHST), Sungkyunkwan University, Seoul, Korea; Cell and Gene Therapy Institute, Samsung Medical Center, Seoul, Korea; Department of Pediatrics, Samsung Medical Center, Sungkyunkwan University School of Medicine, Seoul, Korea; Cell and Gene Therapy Institute, Samsung Medical Center, Seoul, Korea; Department of Pediatrics, Samsung Medical Center, Sungkyunkwan University School of Medicine, Seoul, Korea; Cell and Gene Therapy Institute, Samsung Medical Center, Seoul, Korea; Department of Pediatrics, Samsung Medical Center, Sungkyunkwan University School of Medicine, Seoul, Korea; Cell and Gene Therapy Institute, Samsung Medical Center, Seoul, Korea; Department of Pediatrics, Gangnam Cha Hospital, Cha University, Seoul, Korea

**Keywords:** cell therapy, mesenchymal stromal cells, diseases, neonatal, bronchopulmonary dysplasia, intraventricular hemorrhage, hypoxic ischemic encephalopathy, translational medicine

## Abstract

Despite recent advances in neonatal intensive care medicine, neonatal disorders such as (bronchopulmonary dysplasia [BPD], intraventricular hemorrhage [IVH], and hypoxic ischemic encephalopathy [HIE]) remain major causes of death and morbidity in survivors, with few effective treatments being available. Recent preclinical studies have demonstrated the pleiotropic host injury-responsive paracrine protective effects of cell therapy especially with mesenchymal stromal cells (MSCs) against BPD, IVH, and HIE. These findings suggest that MSCs therapy might emerge as a novel therapeutic modality for these currently devastating neonatal disorders with complex multifactorial etiologies. Although early-phase clinical trials suggest their safety and feasibility, their clinical therapeutic benefits have not yet been proven. Therefore, based on currently available preclinical research and clinical trial data, we focus on critical issues that need to be addressed for future successful clinical trials and eventual clinical translation such as selecting the right patient and optimal cell type, route, dose, and timing of MSCs therapy for neonatal disorders such as BPD, HIE, and IVH.

## Graphical Abstract

**Graphical Abstract:**
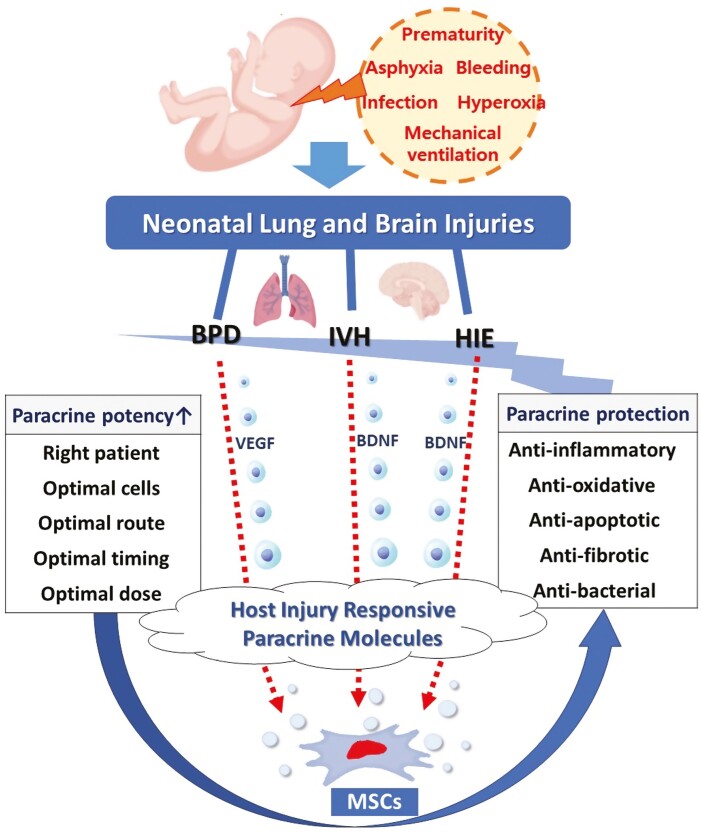

**Significance statement**

Cell therapy, especially with mesenchymal stromal cells (MSCs), acts as a factory for paracrine factors, exhibiting pleiotropic protective effects including immune-modulatory, anti-oxidative, anti-inflammatory, anti-apoptotic, anti-fibrotic, and/or anti-bacterial properties, and facilitating the regeneration of damaged host tissues. These effects align with the complex etiology of neonatal disorders such as bronchopulmonary dysplasia (BPD), intraventricular hemorrhage (IVH), and hypoxic ischemic encephalopathy (HIE). Advances in preclinical and clinical research have driven the development of MSCs therapy for effective applications in neonatal medicine. Successful clinical translation requires resolving issues, such as suitable cell type selection, precise indications, timing, administration routes, and dosages. A methodical and dedicated approach to overcome these challenges is essential to establish cell therapy for treating neonatal disorders such as BPD, IVH, and HIE.

## Introduction

Despite recent progress in neonatal intensive care medicine, neonatal disorders such as bronchopulmonary dysplasia (BPD), intraventricular hemorrhage (IVH), and hypoxic ischemic encephalopathy (HIE) persist as significant contributors to mortality and serious morbidities in neonates. With limited effective treatments for these disorders, the development of new, safe, and effective therapies is an urgent issue.

Recently, various preclinical studies have demonstrated the benefits of stem cell therapy using a wide range and source of cells for attenuating host tissue injury in newborn animal models of BPD,^1-4^ HIE,^5,6^ and IVH.^7^ Here, we choose to highlight mesenchymal stromal cells (MSCs) as a source of cell therapies for neonatal conditions and unique trends/recommendations emerging from research as MSCs are the most extensively studied cell type, owing to their widespread distribution throughout the body, accessibility, and the absence of ethical or safety concerns.

Although various early-phase clinical trials have suggested safety and feasibility, clinical trials showing the therapeutic benefits of MSCs therapy are yet lacking.^2-18^ We focus on the critical issues, including optimizing MSCs delivery to the appropriate patients through the optimal route, at the right timing and dose. These considerations are imperative for successful clinical trials and the clinical translation of MSCs therapies for neonatal disorders in the near future. Subsequent sections will discuss future perspectives based on current preclinical research and clinical trial data, addressing each critical issue in detail.

## Rationales for use of MSCs therapies for various neonatal disorders

Unlike any other single therapeutic agent controlling only one aspect, the pleiotropic beneficial immune-modulatory, anti-inflammatory, anti-apoptotic, anti-oxidative, and anti-fibrotic effects of MSCs therapy have been observed in the newborn animal studies of BPD,^1-4^ HIE,^5,6^ and IVH.^7^ These findings suggest that MSCs therapy could be a new promising therapeutic candidate for various neonatal disorders with complex multifactorial etiologies.

No long-term graft survival^19^ and the protective effects mediated by not only MSCs but also their conditioned media^20^ and extracellular vesicles (EVs)^21,22^ suggest that the protective mechanisms of MSCs therapy are predominantly mediated by paracrine action through the secretion of biological factors/EVs^2-4,23-26^ rather than by their direct regenerative action.^27,28^ The cell-free MSCs-derived EVs possessing similar protective and reparative properties to their parental cells might represent a promising new therapeutic approach as they could circumvent the obstacles and risks associated with the use of live stem cells.^2-4,6,22,24,25^ Moreover, variable key paracrine factors secreted by the same source of MSCs mediating their beneficial effects in different animal disease models of BPD and IVH suggest that there is a crosstalk and interplay between the graft and injured host tissue site, and the MSCs act as a “paracrine factors factory” that sense the local micro-environmental noxious stimuli, and secrete variable host injury responsive key paracrine factors with pleiotropic beneficial effects to enhance the repair and regeneration of the damaged host tissue.^19,25,27^

## Strategies to enhance therapeutic efficacy of MSCs therapy

Given that paracrine potency of the MSCs is a major determinant of their therapeutic efficacy, the following strategies to enhance the paracrine potency of MSCs might improve their ensuing therapeutic efficacy.

### Delivering the ideal cells

#### Source of MSCs matters

MSCs are inherently heterogenic due to their crude definitions, and the paracrine potency and the ensuing therapeutic efficacy of MSCs might vary according to their sources.^2,3^ The use of gestational tissues such as umbilical cord (UC) or umbilical cord blood (UCB) derived MSCs, usually discarded at birth, is attractive due to their easy availability and the absence of ethical concerns. Moreover, compared to adult tissues such as adipose tissue (AT) or bone marrow-derived MSCs, the UCB-derived MSCs exhibit reduced immunogenicity,^29^ higher proliferative capacity and paracrine potency in vitro, and enhanced in vivo paracrine potency and therapeutic efficacy for attenuating hyperoxic lung injuries in newborn rats. The tissue source and donor age can negatively impact the paracrine potency and therapeutic efficacy of MSCs. Therefore, gestational tissues are the most ideal sources of MSCs in clinical setting.^2,3,6^

#### Autologous versus allogeneic MSCs Therapy

There are pros and cons of autologous versus allogeneic MSCs therapy.^2,30^ While the patient-derived autologous MSCs are safe and immunologically compatible, the time-consuming and costly processes involved in isolating and expanding autologous MSCs, and the risk of carrying potential disease candidate genes might be their disadvantages. Although healthy donor-derived allogeneic MSCs are at risk for immunological rejection and elimination due to the major histocompatibility complex (MHC) I antigen as well as minor antigens, they are immune-privileged owing to their lack of the MHC II antigen and their capability to inhibit the proliferation and function of immune cells.^2,14^ Thus far, no administration-related adverse events have been observed in recipients of MSCs among newborns without co-immunosuppression.^12,13^ Furthermore, considering the advantages of allogeneic MSCs therapy, including the less costly large-scale production of pre-selected best quality MSCs than autologous MSCs, and the production of “off-the-shelf” allogeneic MSCs that are readily available to the patient whenever they are clinically indicated, the current trend is toward the clinical use of ready to use allogeneic cell products.

#### Standardization of manufacturing MSCs (regulatory issues)

Despite expanded MSCs research during last 50 years, only 13 MSCs products have been so far market authorized in a few countries including 6 in the United States and 5 in Korea.^25,31^ While the regulatory framework might differ across countries, the following regulatory requirements must be met for successful clinical translation.^25,31,32^ For allogeneic MSCs, donor screening would be necessary to minimize the risk of communicable disorders transmission, and the guidance on good tissue products could provide safeguards to prevent contamination during the manufacturing process.^31,33^ Standardizing isolation and expansion procedures, minimizing batch variability, enhancing cell potency, and large-scale manufacturing of high-quality clinical-grade products under good manufacturing practice (GMP) are essential considerations for the successful application of MSCs.^34^ Interestingly, the risk of malignant transformation and senescence increases with the passage which is essential for expansion in vitro and large-scale production of MSCs.^16^ Although observing karyotype stability and no senescence up to the 11th passage of human UCB-derived MSCs, we opted for passage 6 MSCs in clinical trials for BPD and IVH to mitigate these potential risks.^16,33^ Notably, allogeneic human UCB-derived MSCs manufactured in strict compliance with GMP criteria and approved by both the Korean and American Food and Drug Administration (FDA) have been used in clinical trials for BPD^15,16,18^ and IVH.^17^

#### Paracrine potency assay

Paracrine potency assay measuring the biological/biochemical activities of MSCs that represent their mechanism of action could circumvent the donor heterogeneity and lot variability of MSCs, and could thus select the MSCs with best clinical therapeutic efficacy.^2,3,35^ Boregowda et al reported the transcription factor Twist1-level-dependent variations of MSCs for mediating their angiogenic, anti-inflammatory, and immunomodulatory activities, and thereby developed a Twist1 expression level of MSCs dependent Clinical Indication Prediction Scale suitable for matching the batches of MSCs to different disease indications.^36^

In hyperoxic lung injury of newborn rats, the extent of VEGF gene expression measured by microanalysis,^37^ and the VEGF levels measured by ELISA^38^ were associated with the attenuation of hyperoxia-induced impaired alveolarization and angiogenesis of MSCs as well as their anti-oxidative, anti-inflammatory, anti-apoptotic and immune-modulatory effects both in vitro and in vivo, and the protective effects of MSCs against hyperoxic lung injuries both in vitro and in vivo were abolished with co-administration of anti-VEGF antibody or VEGF knockdown in MSCs by transfection with small interfering (si)RNA specific for human VEGF.^39^

In newborn rats with severe IVH, significant upregulation of BDNF in MSCs in both DNA and antibody microarrays was associated with the attenuation of PHH, impaired behavioral tests, increased astrogliosis, apoptosis, inflammatory cytokines, and decreased myelin basic protein, and with BDNF knockdown in MSCs with human BDNF siRNA transfection, the protective effects of MSCs against neonatal severe IVH were abolished.^40^ In neonatal HIE, genetically engineered overexpression of BDNF in MSCs significantly enhanced their protective effects against severe HIE both in vitro and in vivo.^41^ Our preclinical data suggest that measuring VEGF or BDNF levels in MSCs could be a surrogate marker for selecting the MSCs with best paracrine potency and the ensuing clinical therapeutic efficacy for BPD and HIE/IVH, respectively.

#### Preconditioning of MSCs

Recent studies have shown that in vitro preconditioning such as exposure to hypoxia, lipopolysaccharides, growth factors, and pharmacological/chemical agents enhances the paracrine potency and the therapeutic efficacy of MSCs. The paracrine profiles of the secretome obtained from preconditioned MSCs varied according to the regimens used. Previously, we compared various preconditioning regimens of MSCs, and observed thrombin preconditioning best accelerated skin wound healing compared with hypoxia, lipopolysaccharide, or H_2_O_2_ preconditioning by boosting EV biogenesis and enriching their cargo secretome contents.^42^ Thrombin preconditioning significantly boosted the neuroprotective effects of naïve MSCs against severe HIE^43^ and IVH^44^ by maximizing their paracrine, anti-inflammatory, anti-astroglial, and anti-apoptotic effects. Recently, the Korean FDA approved a phase I clinical trial involving thrombin-preconditioned UC-derived MSCs administered to treat HIE and IVH (KCT0004851).

#### Genetic engineering of MSCs

There is a lot of research now investigating induced pluripotent stem cell (iPSC)-derived MSCs to overcome the current limitations of MSCs such as their limited proliferative capacity and heterogeneity in paracrine potency and therapeutic efficacy.^25,45^ Moreover, the enhancement of a specific paracrine factor release by iPSC-derived MSCs through genetic overexpression represents a promising therapeutic strategy.^25,46^ In newborn rats with severe HIE, BDNF-overexpressing immortalized MSCs have demonstrated >10-fold hypersecretion of BDNF and > 5-fold more effective than naïve MSCs for attenuating oxygen glucose deprivation induced oxidative stress and cytotoxicity in vitro, and only the BDNF overexpressing MSCs but not the naïve MSCs significantly attenuated the in vivo HIE induced severe brain injuries.^41^ After their low-dose gamma irradiation, no tumorigenicity was observed in the nude mice. Overall, genetically engineered iPSC-derived MSCs showing better paracrine potency and therapeutic efficacy might represent a better therapeutic strategy that could overcome the major drawbacks of MSCs such as their heterogeneity and limited ex vivo expansion.

### Selecting the right patients of MSCs therapy

Despite its potential benefits, cautious risk benefit evaluation must be made for selecting the right patients who might benefit from MSCs therapy. Early identification of the infants at the highest risk of later developing BPD is crucial for the success of MSCs therapy to early rescue/prevent BPD. As young gestational age and prolonged respiratory support are 2 well-known prime predictors of BPD development in preterm infants,^47^ we enrolled extremely preterm infants at 23-28 gestational weeks (GW) requiring prolonged ventilator support with respiratory deterioration at postnatal days 5-14 in our recently conducted phase II double-blind randomized clinical trial.^15^ The sample size of 66 infants was calculated based on our previous phase I clinical trial showing 38% reduction in the BPD/death rate with MSCs administration of 33% compared with 71% of the historical control group.^16^ While significant reduction in severe BPD from 53% to 19% with MSCs therapy was observed in the 23-24 GW sub-group, this study was under-powered to detect its therapeutic efficacy in the total extremely preterm infants at 23-28 GW. The insufficiency of this study to reach statistical significance primarily resulted from much reduced actual 33% than the estimated 71% BPD/death rate in the 25-28 GW control group owing to recent improvements in neonatal intensive care medicine. Accordingly, further studies focusing on the most immature infants at 23-24 GW who are at the highest risk of BPD/death are currently underway (NCT03392467).

For HIE, therapeutic hypothermia is the current standard of care and is known to improve the neurological outcome in 15% of HIE infants, but it is not quite effective especially in severe cases.^6,48^ In the newborn rats with severe HIE involving >50% of the ipsilateral hemisphere volume, we have observed that only combined treatment of hypothermia and MSCs administration rather than either therapy alone significantly attenuated the severe HIE brain injury.^49,50^ Overall, these findings suggest that enrolling only the infants with moderate/severe HIE showing no clinical improvement after hypothermia treatment might be the best enrollment criteria to test the therapeutic efficacy of MSCs against HIE for the upcoming phase II clinical trial.

For experimental IVH, by injecting large 200 μL rather than 100 μL of dam’s blood intraventricularly, we were able to persistently induce severe IVH, and the ensuing progress of post-hemorrhagic hydrocephalus (PHH) and brain injuries in the newborn rats, and using this animal model simulating the clinical course of severe IVH, we have observed significant attenuation of severe IVH induced PHH and brain injuries with MSCs therapy.^51^ Since >50% of preterm infants diagnosed with severe (≥grade 3) IVH^52^ either die or develop PHH in up to 70% of cases,^53^ the preterm infants with severe IVH at the highest risk of death/brain injury might be the best enrollment candidate for future phase II trial testing the therapeutic efficacy of MSCs to prevent the progress of PHH and brain injuries after severe IVH.

### Determining the optimal route of MSCs administration

The essential factor for enhancing host injury-responsive paracrine protective effects is the close proximity of donor cells to the injury site, given that paracrine signals have a limited range.^54^ In experimental BPD, despite 4-fold larger systemic intravenous(IV)/intraperitoneal (IP), (2 × 10^6^) than local intratracheal (IT, 5 × 10^5^) dose of MSCs, more infused MSCs were recovered in the lung tissue with IT than IV/IP along with their better protection against neonatal hyperoxic lung injury.^37,55^ In clinical trials of BPD,^15,16,18^ where all preterm infants requiring MSCs therapy are already intubated for ventilator support, local IT delivery of MSCs was seamlessly incorporated. MSCs were administered in 2 aliquots with no administration-associated side effects were observed. While the first human infant receiving IV infusion of human amnion cells experienced transient cardiorespiratory compromise during cell infusion, consistent with a pulmonary embolic event, no such adverse events were observed in the subsequent 5 infants after changing the cells administration methods including such as introducing an inline filter, reducing the cell concentration and rate of infusion in another phase I clinical trial for BPD.^14^

In experimental IVH, the 5-fold larger IV (5 × 10^5^) than intracerebrocentricular (IC, 1 × 10^5^) dose of MSCs showed equal therapeutic efficacy for protecting against severe IVH.^56^ These findings suggest that the less invasive systemic IV route might be a good alternative route for clinically unstable patients who could not tolerate more invasive IC delivery of MSCs for neonatal neurological disorders. In clinical trials of IVH/HIE, both local IC and systemic IV administration of MSCs proved safe and feasible.^10,17^

Recently, the safety and feasibility of the least invasive intranasal (IN) administration have been reported in a phase I clinical trial for treating perinatal arterial ischemic stroke in neonates.^11^ However, since IN delivery of MSCs showed no protection against a murine model of hyperoxic lung injury,^57^ further placebo controlled phase II clinical trial would be necessary to prove their clinical therapeutic efficacy for neonatal disorders.

### Determining the optimal timing of MSCs administration

Determining the optimal timing of MSCs is a key issue that remains to be clarified. In preclinical studies of BPD, early at postnatal day (P) 3-4 but not late at P10-14 significantly attenuated hyperoxic lung injuries.^58-60^ In clinical trials of BPD,^15,16,18^ we thus have enrolled only the extremely preterm infants at 23-28 GW requiring prolonged ventilator support with respiratory deterioration at P5-14. However, all the infants who received MSCs administration late for established BPD in a case series study^9^ and phase I clinical trial^14^ either died or had complications of BPD. Based on the preclinical and clinical data, the therapeutic time window of MSCs therapy for BPD seems to be narrow, and in the clinical setting, early identification of the highest risk extremely preterm infants and MSCs therapy within the first few days would be more preferable over later administration in the infants with established BPD.

In experimental IVH, the protection of MSCs administration against severe IVH was time dependent showing significant protection only early at P6 peak plateau phase but not late at P10 subsiding phase of inflammation.^61^ In a phase I clinical trial of IVH,^17^ MSCs were thus administered within 7 days of the initial diagnosis of severe IVH in preterm infants.

For HIE, applying hypothermia treatment within 6 hours after HIE is the first step of clinical treatment.^6,48^ Preclinically, hypothermia broadened the therapeutic time window and augmented the neuroprotective effects of MSCs against severe HIE.^49,50^ For our recently conducted phase I clinical trial for HIE (KCT0004851), MSCs were administered within 72 hours following the completion of hypothermia treatment to moderate-severe HIE term newborns showing no clinical improvement. Taken together, in the clinical setting, MSCs therapy as early as possible for early rescue/preventive therapy would be most favorable for their best therapeutic outcomes.

### Determining the optimal dose of MSCs administration

Considering the 4- to 5-fold reduced dose of MSCs with better or equal therapeutic efficacy via local IT or IC route over systemic IV administration in experimental BPD and IVH, respectively, the route of MSCs delivery might be a major determinant of optimal dosing. For BPD, based on our preclinical data showing dose-dependent attenuation of hyperoxic lung injury via IT administration of MSCs in the range of 0.5-50 × 10^6^ cells/kg body weight,^62^ the therapeutic efficacy of 1-2 × 10^7^ cells/kg IT administered MSCs were tested in the phases I and II clinical trials for BPD.^15,16,18^ In a case series study^9^ and phase I clinical trial^14^ for established BPD, 1 × 10^6^ cells/kg and increasing weekly dose for 5 weeks of 1.1-13.9 × 10^6^cells/kg were IV administered without therapeutic efficacy, respectively.

In phase I clinical trials for IVH/HIE, a single IC dose of 0.5-1 × 10^7^ cells/kg was given. For HIE, IV twice dose of 2 × 10^6^ cells/kg at 48 hours and 2 months was delivered. For perinatal arterial ischemic stroke, IN delivery of 45-50 × 10^6^ cells/kg dose was administered.

Since all the phase I clinical trials testing different doses of cells via variable routes were safe and feasible without any dose-limiting toxicity or serious adverse events, based on the preclinical and clinical studies data, determining the optimal dose via optimal route with maximal clinical benefits would be an important task in future clinical trial.

## Conclusions

Unlike traditional drug treatments delivering a single chemical agent at a specific dose, MSCs serve as a “paracrine factors factory” that exert their host injury responsive pleiotropic protective effects. MSCs therapy could thus be a game-changer for treating neonatal disorders such as BPD, IVH, and HIE with complex multifactorial etiology. Recent progress in both preclinical research and clinical trials has brought human MSCs therapy for newborns a step closer to clinical translation. However, further resolution of several issues, including optimal cells, clinical indications, timing, route, and dosage, is necessary for successful clinical translation soon. Proceed step-by-step, rather than in haste, with relentless efforts to overcome these barriers is crucial to make MSCs therapy the next breakthrough for treating neonatal disorders.

## Data Availability

No new data were generated or analyzed in support of this research.
